# Increased heart rate variability but no effect on blood pressure from 8 weeks of hatha yoga – a pilot study

**DOI:** 10.1186/1756-0500-6-59

**Published:** 2013-02-11

**Authors:** Marian E Papp, Petra Lindfors, Niklas Storck, Per E Wändell

**Affiliations:** 1 Centre for Family Medicine, Karolinska Institutet, Alfred Nobels allé 12, Huddinge, SE-14183, Sweden; 2 Department of Psychology, Stockholm University, Frescati hagväg 14, Stockholm, SE-106 91, Sweden; 3 Department of Clinical Physiology, S:t Göran Hospital, Stockholm, SE-112 19, Sweden

**Keywords:** Autonomic balance, Blood pressure, ECG, Heart rate variability, Yoga

## Abstract

**Background:**

Yoga exercises are known to decrease stress and restore autonomic balance. Yet knowledge about the physiological effects of inversion postures is limited. This study aimed to investigate the effects of inversion postures (head below the heart) on blood pressure (BP) and heart rate variability (HRV).

**Methods:**

Twelve healthy women and men took part in an 8-week yoga program (60 min once a week). BP was measured with an automatic Omron mx3 oscillometric monitoring device and HRV with a Holter 24-hour ECG at baseline and 8 weeks after the intervention.

**Results:**

There was no significant effect of inversion postures on BP. Nine out of 12 participants showed a significant increase in HRV (p < 0.05) at night (2 hours) on pNN50% (12.7 ± 12.5 to 18.2 ± 13.3). There were no significant changes in other HRV measures such as NN50, LF, HF, LF/HF ratio, LF normalized units (n.u.), HF n.u. and RMSSD.

**Conclusion:**

Eight weeks of hatha yoga improved HRV significantly which suggests an increased vagal tone and reduced sympathetic activity.

## Background

Yoga is frequently used as a lifestyle intervention to reduce stress and restore autonomic nervous system balance [1]. The National Centre for Complementary and Alternative Medicine (NCCAM) refers to yoga as a “mind-body medicine,” with its use being recommended as a non-pharmacological tool for managing stress [1,2]. Hatha yoga uses psychophysical energy movements including specific body postures (asanas), breathing exercises (pranayama) and concentration exercises (dharana) for the mind [1]. Due to the continuous focus on the body, breathing and mind, yoga is psychophysical in character. Focusing on one’s own breathing while practising yoga body movements, and vice versa, can function as a tool to increase awareness of tension/relaxation states. Thus, awareness of one’s body’s position in space can be used as an effective bio-feedback instrument. Many of the slow movements in yoga are considered related to a natural synchronization between breathing and moving (vinyasa) which, in turn, promotes a slower, deeper and more even paced breathing. This, in turn, induces parasympathetic nerve activity and a feeling of relaxation [3]–[5] which can influence heart rate, blood pressure and breathing pace.

A few studies have shown an effect of yoga on the cardiovascular system. For instance, eight to twelve weeks of yoga for individuals with mild to moderate high blood pressure was as effective as medication for hypertension [6]–[8]. Yoga has been shown to increase parasympathetic dominance and, as such, often reduces blood pressure, heart rate and stress while it improves sleep and calms body and mind [1,2,9]. In studies of aerobic exercise and hatha yoga, yoga was shown to increase Heart Rate Variation (HRV), i.e. the beat-to-beat time variation in heart rate [10]–[13].

HRV measurement is a very sensitive method of detecting changes, for example after an intervention [14]. In healthy individuals, the parasympathetic pathway is active during rest, which is reflected by an increased HRV, while low HRV indicates poor health and a higher sympathetic activity [14,15]. However, strong vagal reactivity (high HRV) is associated with good health [14]. Typically, athletes have a higher HRV than do physically inactive individuals. However, intensive training and overtraining can result in a lower HRV [16]. The effects of yoga on HRV is often similar to that of physical activity [17].

A few studies have shown that acute effects of performing yoga postures increased HRV during the night in healthy individuals [18,19]. Long-term effects from 5 weeks of yoga practice (90 minutes once a week) involve significantly increased RR intervals [20]. Taken together, findings from small-scale studies suggest a greater parasympathetic control. Most of the existing research on the effects of yoga has used a mix of yoga postures and breathing exercises, but little is known about the specific effects of different types of yoga postures. In view of this, the present study set out to investigate the long-term effects of specific yoga postures on BP and HRV, focusing mainly on inversions (head below the heart) and semi-inversions (with a deep breathing pattern). It was hypothesized that healthy individuals participating in an 8-week program of hatha yoga consisting mainly of inversions and semi-inversions would show decreased BP and increased HRV.

## Methods

### Participants and procedure

The study recruited participants from a medium-sized organization within the engineering industry. Invitations to take part in the study were sent through the personnel manager to 794 employees and were targeted to either inactive women and men or women and men planning to commence an active lifestyle. Thirty-two individuals responded and 12 fulfilled the inclusion criteria. None of the participants were taking any anti-hypertensive medicines, though medication for asthma, allergy and high cholesterol was used by some participants.

#### Inclusion criteria

Age 25–60 years, having a good general health, having a lightly elevated blood pressure (no higher than 145/95), new to yoga, not exercising regularly or physically active at medium to high intensity (Borg >13).

#### Exclusion criteria

Age > 60 years, diagnosed with high blood pressure and/or taking blood pressure medication; suffering from or diagnosed with chronic diseases that could potentially impede performing yoga such as eye diseases, depression, burnout, indigestion (reflux) and heartburn, which can affect the performance of inversions, as can recent operations during the previous 6 months. Additionally, we excluded individuals with musculoskeletal injuries in the back and/or neck, or suffering from headaches in the morning or while coughing or sneezing. Also, participants performing physical activities more than twice a month and/or at medium or high intensity (out of breath and sweating, Borg >13) were excluded.

Twenty individuals were excluded due to age (5 were too old), taking medication for high blood pressure (2), recent surgery (1), current treatment for brain tumor (1), infection (1), exercising regularly (5), poor possibilities to participate (3), performing mindfulness (1) and ethical reasons (1).

In all 12 individuals took part in the study but 3 individuals had many artifacts on the Holter recording and these were excluded from further HRV analysis. Consequently, the analysis of blood pressure includes 12 individuals while the HRV data come from 9 individuals.

### Assessments/outcome measures

Individual measurements were carried out at the workplace at baseline, before starting the yoga program, and after 8 weeks of yoga exercise. The participants were instructed not to eat, drink coffee or smoke 2–3 hours before measurement.

#### Blood pressure (BP)

BP and heart rate were measured with an automatic Omron mx3 oscillometric blood pressure monitoring device. BP was measured while sitting up after at least 5 minutes of rest. To maximize relaxation, the arm used for measuring BP was firmly supported by the other arm. BP measurements were performed on both arms, at the upper arm in the position of the heart. BP was measured under the same conditions in all participants, i.e. at the same time during the day, at the same sitting position, no talking, and when the individual was relaxed. Mean BP was computed based on readings obtained from both arms. Machine error/accuracy of Omron mx3 was ±3 mmHg (or 2%) of the reading and for the pulse ±5.

#### ECG Holter Analyzer – heart rate variability (HRV)

An Aria-Delmar Holter Analyzer (Spacelabs Healthcare,WA, USA and United Kingdom), was used to record HRV under 24 hours. The sampling rate was 2048 Hz.

For all participants, HRV was measured during the night, but for practical reasons the Holter analyzer was activated during the day. Data were collected between 02.00 and 04.00 a.m. while participants were asleep to minimize potential effects of confounding factors such as alcohol, nicotine or caffeine.

The difference between each R wave in milliseconds (RR intervals), were calculated. All intervals between adjacent QRS complexes in the Electrocardiogram (ECG) resulting from sinus node depolarisations were defined as NN-intervals [21]. NN50 count denotes the number of pairs of successive NN intervals differing by more than 50 ms in the entire recording/sampling. This is highly correlated with frequency domain measures and recognized to be strongly dependent on vagal tone. The pNN50% is a time domain measure of heart rate variability defined as the number of all NN intervals in which the change in consecutive normal sinus intervals exceeds 50 milliseconds divided by the total number of NN intervals measured (pNN50 = (NN50/n-1)*100%) [21,22]. SDNN is defined as the standard deviation of all NN intervals. RMSSD is defined as the square root of the mean of the sum of the squares of the differences between adjacent NN intervals.

The LF/HF ratio was calculated to assess the sympatho-vagal balance. HF is the power in the high frequency range and reflects efferent vagal activity whereas LF is the power in the low frequency range. LF is considered by some researchers to reflect both sympathetic and parasympathetic modulation while others consider it a measure of vagal withdrawal [21]. LF and HF were expressed as measured in normalized units (n.u) which represents the relative value of each power frequency range component in relation to the total power minus the VLF (very low frequency) component. LF and HF in n.u. emphasize the controlled and balanced activity of the two branches of the autonomic nervous system [21].

Obvious technical artifacts were deleted from the ECG and only segments with normal sinus rhythm were included in the analysis. A text file was constructed using subsequent RR-intervals from Aria Holter and imported to Kubios software (filter settings on medium). Time and frequency domain analyses were calculated using Kubios software. A time series was calculated from the RR intervals using spline interpolation with an interpolation rate of 4 Hz. The linear trend was deleted and a Welch filter applied. The frequency analysis was performed using FFT (Fast Fourier Transform).

#### Hand grip strength

Hand grip strength was measured using an electronic hand dynamometer (Camry model EH101). The difference in diastolic BP before and after a 2-minute static (1/3 of maximum hand grip) hand grip strength test was computed. The hand grip measurement was performed standing upright with the device in the dominant hand, the arm held straight out in front of the chest. No other muscles were engaged but the hands. The grip was statically held at a third of the person’s maximum strength. Since no printout function was available, data were manually recorded by the investigator who looked at the monitor of the device. The BP was measured with the device placed on the non-dominant hand before and after the hand grip test (2 minute intervals). A difference in the diastolic BP between the resting BP and the BP after the hand grip test of at least 10 mm Hg indicated an increased reactivity and function of the sympathetic nervous system [23]. This method has been previously suggested to test the efficiency of the cardiovascular system.

#### Borg scale (ratings of perceived exertion)

The Borg scale [24], with a range from 6–20, is often used to measure physical exertion. A rating of ten indicates a heart rate of around 100 beats per minute (bpm). The Borg scale was used to measure the intensity of the yoga class to avoid too high exertion.

#### Waist-hip ratio

Waist-hip ratio (WHR) was used as a complementary measure of body-mass index (BMI) to facilitate detection of cardiovascular diseases among participants. WHR has been used as a tool to measure the degree of obesity [25]. Waist circumference was measured by placing the measuring tape in a horizontal plane midway between the lower rib margin and the hip bone. The hip measurement was taken at the widest point between the two bony prominences at the front of the hips. The same procedure was followed throughout the study.

#### Heart rate

Heart rate measurements were taken using an automatic blood pressure monitoring device and by reading ECG profiles.

### Intervention

The yoga program was 60 minutes long and standardized and was performed in the same manner every time. The program included general poses, inversions and semi-inversions. The order of the postures was the following: cat/cow, shoulder rolls, upper body rotation in cat position, bridge pose (also on one leg), cobra, wall dog variation, wall dog moving down on the wall, down dog, half hand stand towards the wall, chest opener – lying on a roll placed under the rib cage, twisted side angle pose with the knee on the floor, shoulder stand variation to the wall, universal pose, waterfall pose and relaxing lying on the back (for 5–8 minutes) (Additional file 1). As the intervention proceeded, the time spent on inverted poses gradually increased while the time spent on other poses decreased. The total inversion time for each participant during the last 4 weeks of the intervention was around 15-20 minutes. All participants were encouraged to practice at home between classes. If participants had little time they were encouraged to practise only the inverted poses. All classes were run by an experienced certified yoga instructor (U.H.) and all classes took place at the same location, on the same day of the week and at the same time in the afternoon.

A breathing frequency at a rate of 0.1 Hz (6 breaths/minute) was encouraged but many of the participants found it hard to breathe at this speed. The majority were breathing at a rate of 0.2 Hz (12 breaths per minute) though this was not measured directly and is only a rough estimate. All participants had a 30-min assessment at their workplace before and after the intervention. The Regional Ethical Review Board in Stockholm (DNR: 2011/248-31/1) approved the study and all participants signed informed consent forms.

### Data extraction and statistical analysis

#### Statistical analysis

The Kubios HRV analysis program, Version 2.0 from Department of Physics, University of Kuopio, Kuopio, Finland, was used for the ECG analysis. The analysis was carried out at the physiology laboratory at S:t Göran hospital in Stockholm in cooperation with their doctors and the chief executive of the laboratory (N.S). Data from three individuals were excluded from the analysis due to artefacts caused by faulty device operation (2) and electric interference (1).

Wilcoxon’s paired signed-rank tests were used to compare data collected before and after the intervention. It is a nonparametric statistical test applicable to repeated measures on individuals belonging to a small sample.

Effect size (ES) (Cohen’s *d*) was used to measure change, with an ES of 0.20 regarded as a small change, an ES of 0.50 as a moderate change and an ES of 0.80 as a large change [26].

All statistical analyses were performed using MATLAB.

##### Power analysis

We performed both two-tailed and one-tailed post-hoc test, the latter as our hypothesis was that yoga postures could increase HRV. Post –hoc analyses used the program: STATA/IC 11.2.

Post-hoc  statistical power alpha = 0.05, N = 9 *(two-tailed*)

RR mean 0.07, RR triangular index (triang) 0.10, SDNN 0.09, NN50 0.20, pNN50% 0.21, RMSSD 0.12

Post-hoc statistical power alpha = 0.05, N = 9 (*one-tailed*)

RR mean 0.12, RR triang 0.16, SDNN 0.16, NN50 0.32, pNN50% 0.33, RMSSD 0.19

From Table 1: Post-hoc statistical power alpha = 0.05, N = 9 (*two-tailed*)

**Table 1:** **Frequency domain power (ms**^**2**^**) – (FFT spectrum) ECG data and HRV (heart rate variability) in study participants at night (02.00-04.00 am; n = 9)**

	**Before/Baseline**	**After 8 weeks’ yoga**	**P-value**	**Effect size**	**Confidence interval before**	**Confidence interval after 8 weeks**
LF [ms^2^]	1237.9 ± 1072.6 Md 852	1335.2 ± 989.1 Md 1346	0.82	0.10	412-2064	574-2097
HF [ms^2^]	560.7 ± 509.0 Md 454	689.2 ± 505.2 Md 666	0.50	0.27	169-953	300-1078
LF/HF ratio	2.5 ± 1.0 Md 2.2	2.4 ± 1.2 Md 2.3	0.36	0.10	1.7-3.3	1.5-3.3
LF n.u.	69.8 ± 7.9 Md 68.5	66.5 ± 13.1 Md 69.5	0.29	0.32	63.7-75.9	56.4-76.6
HF n.u.	30.2 ± 7.9 Md 31.5	33.5 ± 13.1 Md 30.5	0.29	0.32	24.1-36.3	23.4-43.6

Data are expressed as means ± SD and, when stated, median.

LF = Power in low frequency range (0.04-0.15 Hz). Some researchers believe this involves both sympathetic and parasympathetic modulation (sympathetic involves the blood vessels whereas parasympathetic involves the heart). LF measures withdrawal of vagal tone (Goldstein).

HF = Power in high frequency range (0.15-0.4 Hz), indicates efferent vagal activity.

LF/HF ratio = Ratio is correlated with sympatho-vagal balance.

LF n.u = Represents the relative value of LF power component in proportion to the total power minus the VLF (very low frequency) component in normalized units (n.u).

HF n.u. = Represents the relative value of HF power component in proportion to the total power minus the VLF (very low frequency) component.

n.u. = normalized units.

Confidence interval = 95%.

Md = Median.

LF 0.06, HF 0.11, LF/HF ratio 0.06, LF n.u 0.13, HF n.u 0.13

Post-hoc statistical power alpha = 0.05, N = 9 (*one-tailed*)

LF 0.08, HF 0.18, LF/HF ratio 0.08, LF n.u 0.22, HF n.u 0.22

## Results

Table 2 shows sample characteristics.

**Table 2:** Parameters in whole group (n = 12) and those with ECG measurements (n = 9)

**Parameters**	**Before/Baseline (n = 12)**	**After 8 weeks’ yoga (n =12)**	**P-value (ES)**	**Before/Baseline (n = 9)**	**After 8 weeks’ yoga (n = 9)**	***P*-value (ES)**
Age (y)	49.6 ± 6.2	-	-	50.1 ± 4.8	-	-
Height (m)	1.76 ± 0.08			1.77 ± 0.09		-
Male	8			6		
Women	4			3		
Weight (kg)	80.0 ± 11.9	80.6 ± 12.0	0.22	84.9 ± 11.9	85.4 ± 12.1	0.39
Maximal Handgrip/kg	36.6 ± 8.6	39.0 ± 10.0	0.12 (ES: 0.28)	38.1 ± 9.4	42.5 ± 10.8	0.02* (ES: 0.46)
Waist : hip ratio	0.87 ± 0.08	0.87 ± 0.08	1.0	0.88 ± 0.08	0.88 ± 0.08	0.67
BMI (kg/m^2^)	26.0 ± 3.7	26.2 ± 3.8	0.23	27.1 ± 3.6	27.3 ± 3.9	0.38
BP mean from left and right arm Systolic/Diastolic (mm Hg)	123.3 ± 12.4 79.8 ± 6.3 Md 119.3 80.8	124.1 ± 11.7 82.2 ± 7.6 Md 120.0 83.0	0.62 0.18	123.3 ± 13.2 79 ± 7.4 Md 118.5 75.0	125.8 ± 13.3 83.9 ± 10.7 Md 120.5 83.0	0.89 0.21
Heart rate from Blood Pressure cuff (bpm)	66.5 ± 8.4 Md 66.0	67.4 ± 11.0 Md 64.3	0.83	63.1 ± 4.9 Md 62.0	68.4 ± 12.1 Md 62.0	0.20
Heart rate (from ECG) (n = 10) (bpm)	61.0 ± 6.2 (n = 10) Md (n = 10) 60.5	62.0 ± 10.0(n = 10) Md (n = 10) 62.3	0.77	61.4 ± 6.4 Md 61.0	61.0 ± 10.0 Md 57.3	0.91

Data are expressed as means ± SD and, when stated, median.

Y = years, m = meters, bpm = beats per minute, ES = effect size (change/sd).

* = *p* < 0.05 significant.

Effect size noted when of interest.

Md = Median.

### Blood pressure

There was no significant difference in blood pressure between baseline and follow-up (Table 2). In addition, there were no significant effects on pulse pressure and mean arterial pressure after 8 weeks of yoga.

### ECG Holter analysis – heart rate variability (HRV)

There was a medium (ES 0.45) but significant effect of yoga on pNN50% (mean 12.7 ± 12.5 to 18.2 ± 13.3; Table 3). NN50 also increased. The LF/HF ratio showed a slight but not significant decrease (Table 1). Additionally, analyses of LF and HF showed a trend towards a higher HF indicating an increased vagal tone. No statistically significant differences were found for SDNN, LF n.u. (normalized units) or HF n.u. RMSSD (an estimate of short-term components of HRV data).

**Table 3:** Time Domain Units – ECG data and HRV (heart rate variability) in study participants at night (02.00-04.00 am) (n = 9)

	**Before/Baseline**	**After 8 weeks’ yoga**	**P-value**	**Effect size**	**Confidence interval before**	**Confidence interval after 8 weeks**
RR mean [ms]	996.2 ± 105.4 Md 992.3	1019.8 ± 172.8 Md 1057.1	0.82	0.17	915-1077	887-1153
RR triangular index	16.3 ± 4.5 Md18.0	17.5 ± 5.8 Md 17.9	0.50	0.25	12.8-19.7	13.0-22.0
SDNN (stdRR) [ms]	85.6 ± 21.9 Md 83.4	92.7 ± 39.6 Md 95.6	0.57	0.24	68.7-102.5	62.2-123.2
NN50 count	868.4 ± 823.8 Md 731	1208.7 ± 810.0 Md 1497	0.13	0.44	234-1503	585-1832
pNN50 [%]	12.7 ± 12.5 Md 10.6	18.2 ± 13.3 Md 17.1	*0.035	0.45	3.1-22.3	8.0-28.4
RMSSD [ms]	42.1 ± 19.4 Md 41.0	47.6 ± 21.2 Md 50.2	0.30	0.29	27.2-57.0	31.3-63.9

Data are expressed as means ± SD and, when stated, median. ms = milli seconds.

* = P < 0.05 significant.

NN = RR.

RR triangular index = Total number of all NN intervals divided by the height of the histogram of all NN intervals measured on a discrete scale with bins of 7.8125 ms (1/128 s) [21].

SDNN = the standard deviation of all NN intervals.

NN50 count = Number of pairs of adjacent NN intervals differing by more than 50 ms in the entire recording. This is highly correlated with frequency domain measures and recognized to be strongly dependent on vagal tone.

pNN50% = NN50 count divided by the total number of all NN intervals.

RMSSD = the square root of the mean of the sum of the squares of differences between adjacent NN intervals.

Confidence interval = 95%.

Md = Median.

### Hand grip strength

No significant effects of yoga on hand grip strength were found after analysing the differences in diastolic pressure before and after the hand grip test. However, and as presented in Table 2, analysis of ECG data that were available for nine participants showed a significant increase in maximal hand grip strength (p < 0.02) (Table 2) with an ES of 0.46.

### Borg scale (perceived exertion)

The perceived exertion during yoga class was 12.9 on the Borg scale for the whole group and 12.8 for the ECG group. The intensity was at a comfortable level (<13) for all participants, and many of them reported lower levels of exertion towards the end of the study

### Anthropometric measures

#### Waist-hip ratio

No significant effect of yoga was found on waist-hip ratio (WHR), weight or body mass index.

#### Heart rate

There were no significant changes in heart rate after 8 weeks of yoga (Table 2).

#### Attendance rate (of a total of 8 sessions) and home practice

The attendance rate during the whole intervention was 5.5 times (69%) for the whole group. and 6.0 times (75%) for the ECG group. The total number of home practice sessions was 5.2 during 8 weeks for the whole group and 4.2 for the ECG group.

## Discussion

This study of an 8-week hatha yoga program showed no significant effect on BP but showed a significant medium effect (ES 0.45) of yoga on HRV, with a significant increase in pNN50% (12.7 ± 12.5 to 18.2 ± 13.3). In addition, other measures improved i.e. the NN50, and the HF power component increased, and the LF/HF ratio decreased, but failed to reach statistical significance.

Few other studies have looked into the effects of yoga postures (including many inversions) on HRV in healthy individuals. Specifically, previous findings show that acute effects include increased HRV at night after having practiced yoga [18,19]. Long-term effects include significantly increased RR intervals [20]. Findings from previous small-scale studies have suggested that yoga postures and yogic breathing exercises significantly increase cardiac vagal modulation which, in turn, suggests a greater parasympathetic control.

Despite previous studies showing significant effects on BP after 3-8 weeks of yoga in hypertensive individuals [6,8], no such effects were observed in the current study. This was probably due to participants in the current study being normotensive, i.e. their blood pressure was low or normal already at baseline. Consequently, large changes in blood pressure were not to be expected. Nevertheless, the current findings are in line with previous research showing that 15 minutes of yoga postures had no effect on blood pressure but instead had a significant effect on HRV, on SDNN (square root of variance) [27]. As regards SDNN comparisons with previous research, the recordings must be of the same duration. In the current study, the SDNN recordings were 2 hours long whereas the recordings in an earlier study were 15 minutes long which means that data from the two studies are incomparable [21].

HRV is a very sensitive measure and the current study showed significant effects of yoga on pNN50% at night. High HRV indicates greater parasympathetic control [28] and the physiological adaptations to yoga exercises were similar to those of conventional exercise [13,17]. This was the rationale behind the exclusion of individuals who exercised regularly from the current study. Since this study focuses on long- term effects of yoga on HRV after yoga performance, HRV data both during sleep and yoga practice are not presented. Also, all participants did not have the Holter monitor on while practicing yoga. It is well known that exercise and stretching decreases HRV [29] which means that the measurement of HRV during physical activity would give no valuable information of about long term effects of yoga on HRV.

The current study findings showing a significant effect of yoga on HRV (pNN50) at night are consistent with those of previous research [18]. However, the proportions differ, which may result from our recordings being scheduled during the deepest sleep and during one period (02.00–04.00 a.m.), but also from the fact that our total recording time was 1 hour longer than that of Patra [18]. However, differences in initial pNN50 values are also due to the characteristics of the participants of the current study with the present study including a group of middle-aged healthy participants. The normal number of intervals with a difference of more than 50 ms is approximately 200 intervals per/hour during the day and around 400 intervals per hour at night [14,22]. In this study, an overall increase in pNN50% was detected at night by the end of the intervention. Other studies have had shorter HRV recording times than the 2-hour recording time in the current study. However, as there were not enough Holter devices for all participants to use during the exactly the same time period, the recording times may have differed slightly. Consequently, HRV data cannot be compared during the whole 24-hour period. However, we are not aware of any yoga intervention studies or other interventions presenting full 24-hour HRV data. Here, data collected from HRV recordings during night-time when participants were asleep and wearing the Holter device and thus in a comparable condition, were included in the analysis.

Physical activity have been found important for achieving a high HRV [30]. This was the rationale for choosing to include only inactive individuals who had not practised yoga before in our study. The program studied here was different from cyclic meditation (which involves no inversions and is performed with the eyes closed) used in an earlier study [18]. Yet, this study [18] is one of the few HRV studies showing findings that are comparable to the present results.

Another study [31] showed increased pNN50% during rest in athletes (body builders) who performed 15 minutes of daily stretching for 28 days. However, the pNN50% values were calculated using a heart rate monitor which is not as reliable a device as is the Holter analysis, since the heart monitor often fails to record all true beats from the sinus node. Measures taken after stretching typically show a rapid increase in parasympathetic activity and a lower heart rate, and many yogic postures include a stretching component [29]. This may be one explanation for feelings of relaxation and increased parasympathetic activity after yoga exercises. Thus, yoga may enhance the plasticity of the autonomic nervous system and improve the ability to recover after stress [18,31]. Some studies have shown that yoga lowered the resting heart rate [32], indicating a vagal dominance [18,31]. However, no such effect on heart rate was found in the current study.

Other studies have found a decreased LF/HF ratio (low frequency/high frequency) after yoga, indicating a switch towards vagal dominance. In the present study, the LF/HF ratio decreased but this change was not significant [11,12,18] (Table 1). An increased LF/HF ratio is often seen in older age [30] but has also been related to depression and stress [33].

The present findings show an increased LF power, which measures the parasympathetic withdrawal, but also an increased HF power indicating a higher vagal tone. However, none of these changes were statistically significant. LF and HF were also analysed in normalized units (n.u.) but no significant effects emerged. These findings were consistent with those of earlier studies showing an LF n.u. decrease and an HF n.u. increase following a yoga intervention [18].

RMSSD is an estimate of short-term components of HRV data and was found to increase but not significantly. This trend suggests an increased HRV after the 8-week yoga intervention but the non-significant effect may have been due to the small sample size.

Supine and inverted body postures stimulate the baroreceptor reflex (from altered negative pressure in the upper body) and may create a parasympathetic (vagal) activity [8,34]–[36] while upright postures inhibit it [34]. The baroreceptor reflex which regulates heart rate is closely linked to the parasympathetic nervous system [14,37].

The increased HRV after stretching may be related to the release of vasodilative agents (EDRF = Endothelium-derived relaxing factor) which reduces muscle tone, but could also result from a general systemic psychic-physical relaxation [31].

Some findings suggest that atrial arrhythmia can be restored after an inversion program [38] and a 40-minute program (with 10-minute intervals of posture change that alternatively stimulate the vagal and the sinus nerve) can be as effective as medication in 50% of patients with atrial arrhythmia. This study used inversions in the intervention program. Other researchers report that the upside down position can treat paroxysmal supraventricular tachycardia [35,36,39] when no other methods, such as medication and manual stimulation of the vagus nerve, work. Tai reported a case study where a woman with arrhythmia was able to restore a normal sinus rhythm with a 20-second hand stand when none of the conventional methods worked. According to the researchers the mechanism may involve vagal stimulation due to increased carotid sinus pressure that may possibly restore the baroreceptor reflex function [2,35,36,39,40].

Patients with essential hypertension seem to be able to restore the baroreflex mechanism with lowering of the blood pressure (29 units systolic and 17 units diastolic) after 3 weeks of yoga postures (also inversions) [8]. The inversions may reactivate the malfunctioning baroreflex mechanism by alternating the pressure on the baroreceptors. The baroreflex arc does not function properly in hypertensive, ageing, stressed, inactive and depressed individuals [8,14,33,41,42].

Breathing frequency affects HRV, and respiratory sinus arrhythmia (RSA) has its maximal amplitude with a frequency of 6 breaths per minute; baroreflex sensitivity is also enhanced with this breathing frequency [4]. There is an increased RSA in supine posture [14] and perhaps even more so in an inverted posture [2,14] which might have created a slower breathing frequency among the participants in our study who performed many inversions.

In this study, breathing frequency was not measured but deep breathing was encouraged, and this may have lowered the breathing frequency. There are studies showing a slower breathing frequency and resting heart rate after a yoga intervention [32]. Hyperventilation is common in hypertension, and inhibition of the baroreflex may be a possible mechanism while breathing fast [43] elevates blood pressure.

### Limitations

In this study, the main limitation is the small sample size with a low power. This was due to difficulties in recruiting study participants who were physically inactive. The workplace had a high percentage of physically active employees and consequently the main problem was to find sedentary individuals. This too was the main reason for not having enough participants that could fit into the inclusion criteria and to perform, as initially planned, a randomized controlled trial. Even though the findings showed significant differences between baseline and follow-up, this may have been due to the study being conducted in the spring when the weather allows for more outdoor activities and a more active lifestyle. Also the interaction with the instructor may have created a therapeutic benefit. However, in line with the initial hypothesis, some of the HRV measures increased. With pNN50% being a very sensitive measure, it was not surprising that the increase in pNN50% was statistically significant whereas changes in other HRV measures were not. Some participants were excluded due to the low ECG recording quality. Breathing frequency is an important factor for detecting vagal tone, but this was not measured as participants were instructed to only take deep breaths.

## Conclusions

This small-scale longitudinal pilot study in naive hatha yoga participants, where time spent on inversions increased from 7 minutes to 20 minutes over the eight week period, showed that yoga increased HRV. This suggests that yoga exercises can have a restorative effect on the autonomic nervous system. Some HRV measures, including HF and LF/HF ratio also improved but not significantly. However, larger, randomized controlled studies are needed to confirm the effects of yoga on the sympathetic and parasympathetic nervous system. Future studies should measure breathing frequency, baroreceptor sensitivity and heart rate recovery before, during and immediately after performing different yoga exercises, perhaps focusing solely on inversions or sun salutations to detect associated changes in the autonomic nervous system.

## Competing interests

The authors declare that they have no competing interests.

## Authors’ contributions

MP, PL and PW designed the study, MP performed the study. MP and NS carried out ECG analyses. MP and PW analyzed the results. MP wrote the manuscript assisted by PW and PL, NS was involved in designing the methods and interpreting the results of the ECG analyses. All authors read and approved of the final manuscript.

## Supplementary Material

Additional file 1 **Availability of supporting data. **See appendix A (link) for pictures of the yoga program. The data are stored at CeFAM. Click here for file
