# Supplementary figures and images for: Increased heart rate variability but no effect on blood pressure from 8 weeks of hatha yoga – a pilot study
Source: BMC Res Notes. 2013 Feb 11;6:59. doi: 10.1186/1756-0500-6-59 (PMC3599360; doi:10.1186/1756-0500-6-59)

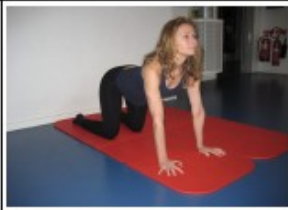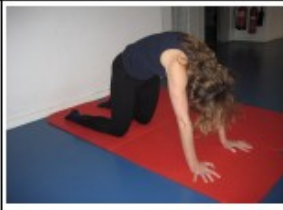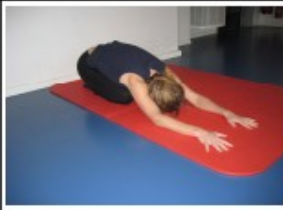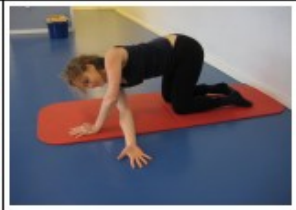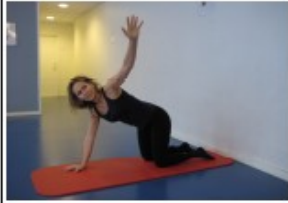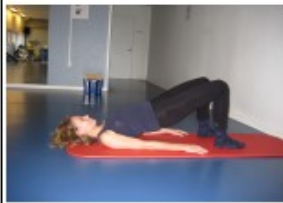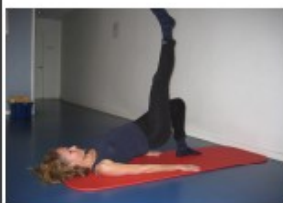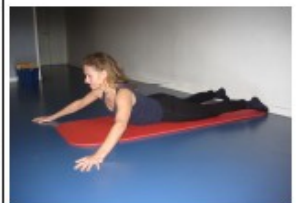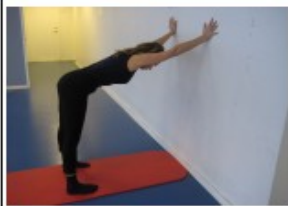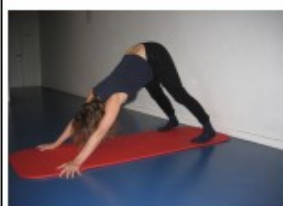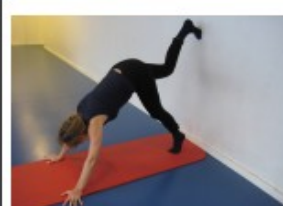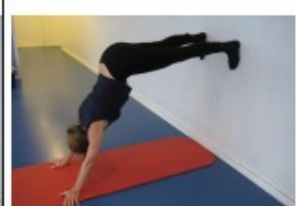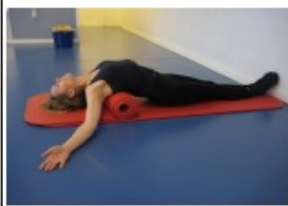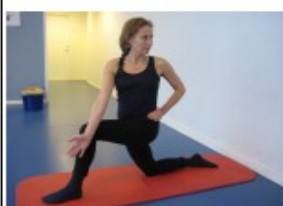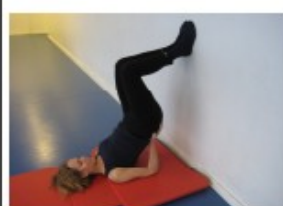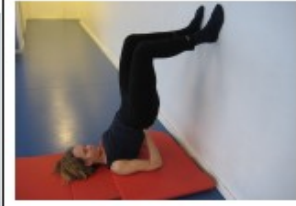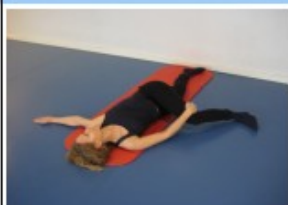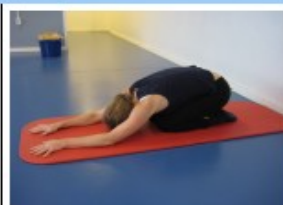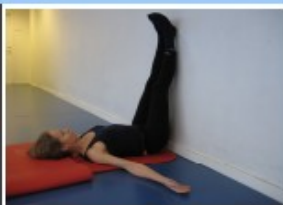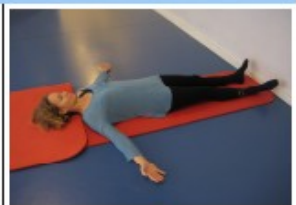

Supplement: Additional file 1 — Availability of supporting data. See appendix A (link) for pictures of the yoga program. The data are stored at CeFAM. [file 1756-0500-6-59-S1.pdf]
